# Survival and surgical outcomes of cardiac cancer of the remnant stomach in comparison with primary cardiac cancer

**DOI:** 10.1186/1477-7819-12-21

**Published:** 2014-01-27

**Authors:** Yi Wang, Chang-Ming Huang, Jia-Bin Wang, Chao-Hui Zheng, Ping Li, Jian-Wei Xie, Jian-Xian Lin, Jun Lu

**Affiliations:** 1 Department of Gastric Surgery, Fujian Medical University Union Hospital, No. 29 Xinquan Road, Fuzhou 350001, Fujian Province, China

**Keywords:** Cardiac cancer of the remnant stomach, Primary cardiac cancer, Prognosis

## Abstract

**Background:**

Although cardiac cancer of the remnant stomach and primary cardiac cancer both occur in the same position, their clinical characteristics and outcomes have not been compared previously. The objective of this study was designed to evaluate the prognosis of cardiac cancer of the remnant stomach in comparison with primary cardiac cancer.

**Methods:**

In this retrospective comparative study, clinical data and prognosis were compared in 48 patients with cardiac cancer of the remnant stomach and 96 patients with primary cardiac cancer who underwent radical resection from January 1995 to June 2007. Clinicopathologic characteristics, survival times, mortality, and complications were analyzed.

**Results:**

The 5-year survival rate was significantly higher in patients with primary cardiac cancer than in those with cardiac cancer of the remnant stomach (28.4% *vs.* 16.7%, *P* = 0.035). Serosal invasion, lymph node metastasis and tumor location were independent prognostic factors for survival. Subgroup analysis, however, showed similar survival rates in patients with primary cardiac cancer and cardiac cancer of the remnant stomach without serosal invasion (25.0% *vs.* 43.8%, *P* = 0.214) and without lymph node metastasis (25.0% *vs.* 38.8%, *P* = 0.255), as well as similar complication rates (20.8% *vs.* 11.5%, *P* = 0.138).

**Conclusion:**

Although the survival rates after radical resection in patients with cardiac cancer of the remnant stomach were poorer than in those with primary cardiac cancer, they were similar in survival rates when patients without serosal invasion or lymph node metastasis. Therefore, early detection is an important way to improve overall survival in cardiac cancer of the remnant stomach.

## Background

Adenocarcinoma of the gastric remnant was first described by Balfour in 1922 [1]. And gastric remnant cancer is defined as a carcinoma detected in the remnant stomach more than 5 years after primary surgery for a benign disease or 10 years after primary surgery for a malignant disease, regardless of the pattern of the initial surgical resection or the type of reconstruction [2,3]. According to the literature, the incidence of gastric remnant cancer ranges between 3% and 10% of all gastric cancer patients [4-6]. Previous studies have evaluated the clinicopathologic characteristics of gastric remnant cancer and compared the surgical results between patients with gastric remnant cancer and those with primary proximal gastric cancer [7-10], but few studies have investigated patients with cardiac cancer of the remnant stomach.

Cardiac cancer of the remnant stomach is defined as a malignant tumor in the gastric remnant located in the cardiac region. Due to a lack of specific symptoms, most patients are initially diagnosed at an advanced stage, resulting in low rates of curative resection and consequently in poor prognosis [11,12]. Both cardiac cancer of the remnant stomach and primary cardiac cancer occur in the upper third of the stomach, but their clinical characteristics and outcomes have not been compared previously. This study analyzed clinical data in 48 patients with cardiac cancer of the remnant stomach and 96 patients with primary cardiac cancer who underwent radical resection from January 1995 to June 2007, in order to evaluate differences in patient prognosis.

## Methods

### Patients

This retrospective comparative study involved 48 patients with cardiac cancer of the remnant stomach and 96 with primary cardiac cancer who underwent curative resection in the Department of Gastric Surgery, Fujian Medical University Union Hospital, between January 1995 and June 2007. For cardiac cancer of the remnant stomach, criteria for inclusion in the study were: (1) clear preoperative diagnosis of gastric cancer by gastroscope biopsy pathological examination; (2) preoperative chest X-ray, abdominal ultrasound, and upper abdominal CT scan, showing no distant metastases to the liver, lung, or abdomen; (3) radical gastrectomy with lymph node dissection carried out and pathological diagnosis recommending R0 resection; and (4) tumor size clearly recorded on surgical records. Exclusion criteria were: (1) the patient not having gastric cancer in the upper part of the stomach; (2) the patient suffering multiple tumors of the stomach; (3) intraoperative tumor peritoneal dissemination observed; (4) patient with history of suffering from other malignant tumor; or (5) pathological diagnosis of the patient not recommending R0 resection.

For primary cardiac cancer, patients were paired by: age (± 5 years); operation time (± 2 years); TNM stage (UICC 7th), including similar depth of invasion (T) and lymph node metastasis (N); lymph node dissection pattern; and postoperative chemotherapy regimens and number of chemotherapy cycles. In addition, all operations were performed by the same surgeon. None of these matching factors differed significantly between the two groups, as shown by paired t tests and chi-square tests (*P* >0.05). The demographic and clinical characteristics of the two groups are shown in Table 1.

**Table 1:** Demographic and clinical characteristics of patients with cardiac cancer of the remnant stomach and primary cardiac cancer

**Characteristic**	**Primary cardiac cancer of remnant stomach**	**Primary cardiac cancer**	***X*** **2/t**	***P *** **value**
Age (years)	61.54 ± 8.84	61.59 ± 8.31	−0.035	0.972
Gender			0.000	1.000
Male	44	88		
Female	4	8		
Borrmann type			−0.954	0.342
I	4	0		
II	14	30		
III	26	62		
IV	4	4		
Tumor size (cm)	5.9 ± 2.2	6.2 ± 3.1	−0.634	0.527
Histological type			−1.223	0.224
Undifferentiated	15	21		
Differentiated	33	75		
Depth of invasion			0.000	1.000
T1	2	4		
T2	6	12		
T3	18	36		
T4a	22	44		
Lymph node metastasis			0.000	1.000
N0	20	40		
N1	10	20		
N2	14	28		
N3	4	8		
Positive lymph nodes (*n*)	2.7 ± 3.8	2.8 ± 4.2	0.000	0.873
Dissected lymph nodes (*n*)	18.0 ± 5.8	26.9 ± 8.9	1.000	0.000
Lymph node dissection pattern			0.000	1.000
D1	6	12		
D2/D2+	42	84		
Combined organ resection	8 (16.7%)	24 (25.0%)	−1.131	0.260

*Analysis of variance. P < 0.05 is significant.

### Surgical approaches

Patients with cardiac cancer of the remnant stomach were all underwent radical remnant gastrectomy with lymph node dissection (R0; include D1 (6 cases) and D2/D2+ (42 cases) dissection); eight (16.7%) cases were combined organ resection, included resection of left lateral hepatic lobe (3 cases), distal pancreas (2 cases), spleen (2 cases), and gall bladder (1 case). Patients with primary cardiac cancer were all underwent radical gastrectomy with lymph node dissection (R0; include D1 (12 cases) and D2/D2+ (84 cases) dissection); 24 (25.0%) cases were combined organ resection, included resection of spleen (9 cases), left lateral hepatic lobe (7 cases), distal pancreas (4 cases), gall bladder (3 cases), and transverse colon (1 case). All cases with cardiac cancer of the stomach in this study were AEG II and AEG III according to the Siewert Classification, and they all underwent radical gastrectomy with lymph node dissection and pathological diagnosis recommending R0 resection according to the guidelines of the Japanese Classification of Gastric Carcinoma (JCGC) [2]. A proximal margin of at least 3 cm is recommended for T2 or deeper tumors with an expansive growth pattern and 5 cm is recommended for those with infiltrative growth pattern. All cases in our study were examined the proximal resection margin by frozen section to ensure an R0 resection. We explained the surgical procedure to the prospective patients, including its advantages and risks, and obtained informed consent before the procedure. All patients were received postoperative chemotherapy, the regimen was mainly platinum and fluorouracil derivatives, number of chemotherapy cycles were ranging from 1 to 6.

### Postoperative follow-up

Patients were followed up by outpatient visits, letters, and telephone calls. Overall survival was calculated from the date of diagnosis until the date of last contact, date of death, or date on which survival information was collected. All surviving patients were followed up for more than 5 years.

### Statistical analysis

Statistical analyses were performed using the SPSS 18.0 statistical software package. Means were compared using t-tests and categorical data using Chi-square tests. Survival rate was calculated according to the Kaplan-Meier method and compared using the log-rank test. A Cox proportional hazards model was constructed for multivariate analysis of prognosis. The correlation between two groups in mortality and postoperative complication rate was analyzed with logistic regression model. *P* values <0.05 were considered statistically significant.

### Ethical approval

Ethics committee of Fujian union hospital approved this retrospective study. Written consent was given by the patients for their information to be stored in the hospital database and used for research.

## Results

### Patient prognosis

**Figure 1:**
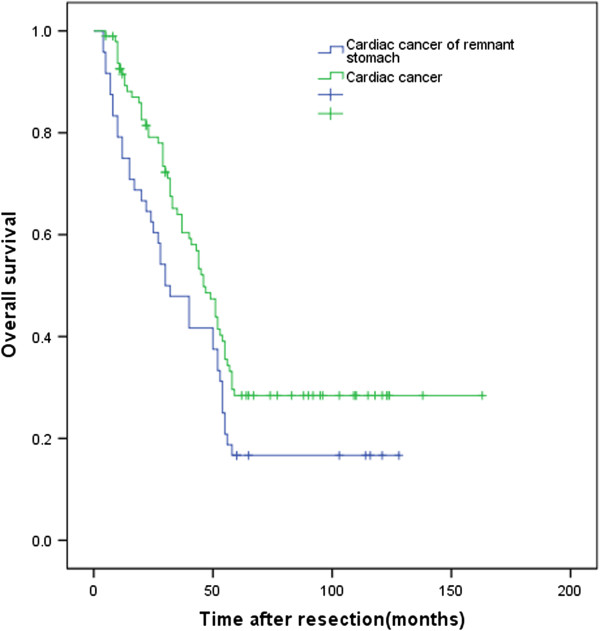
**Kaplan-Meier survival curves for patients with cardiac cancer of the remnant stomach and primary cardiac cancer.***P* = 0.035 (log rank test).

Of the 144 patients with cardiac cancer of the remnant stomach or primary cardiac cancer, 124 (86.1%) were followed up for 4 to 163 months, included 43 (89.6%) patients with cardiac cancer of the remnant stomach and 81 (84.4%) patients with primary cardiac cancer. The postoperative 5-year survival rate in all patients was 24.3%. Median overall survival was 30.0 months (95% confidence interval (CI) 19.0-41.0 months) in patients with cardiac cancer of the remnant stomach and 46.0 months (95% CI 39.5-52.5 months) in patients with primary cardiac cancer. The postoperative 5-year survival rates in these two groups were 16.7% and 28.4%, respectively (*P* = 0.035, Figure 1).

### Univariate analysis of factors prognostic for survival in all patients

Of the nine clinicopathological variables identified by univariate analysis, showed that factors prognostic for survival in all patients were tumor size (*P* = 0.004), serosal invasion (*P* = 0.001), lymph node metastasis (*P* = 0.000), and tumor location (*P* = 0.035) (*P* <0.05 each; Table 2).

**Table 2:** Univariate analysis of factors prognostic for survival in all patients

**Variables**	**Patients ( *n *)**	**5-year survivalrate (%)**	***X*** **2**	***P *** **value**
Gender			0.646	0.422
Male	132	25.1		
Female	12	16.7		
Age (years)			3.794	0.051
≥60	92	19.7		
<60	52	33.1		
Tumor size (cm)			8.175	0.004
≥5	103	21.2		
<5	41	31.8		
Serosal invasion			11.045	0.001
Yes	124	21.5		
No	24	37.5		
Lymph node metastasis			14.552	0.000
Yes	84	17.9		
No	60	33.7		
Histological type			0.936	0.333
Differentiated	108	25.1		
Undifferentiated	36	22.2		
Combined organ resection			0.112	0.737
Yes	32	33.7		
No	112	22.9		
Borrmann type			1.998	0.573
I	4	25.0		
II	44	20.2		
III	88	27.1		
IV	8	12.5		
Tumor location			4.460	0.035
Cardiac region of the remnant stomach	48	16.7		
Cardiac region	96	28.4		

*Analysis of variance. P < 0.05 is significant.

### Multivariate analysis of prognostic factors for survival in all patients

Multivariate analysis of the four factors prognostic for survival, using a Cox’s proportional hazard regression model, showed that serosal invasion (*P* = 0.043), lymph node metastasis (*P* = 0.023), and tumor location (*P* = 0.014) were each independent, statistically significant factors prognostic of survival (*P* <0.05 each; Table 3).

**Table 3:** Multivariate analysis of factors prognostic of survival in all patients

**Variables**	**B**	**SE**	**Wald**	***P***	**RR**	**95% CI**
Tumor size	0.133	0.266	0.251	0.616	1.143	0.678–1.925
Serosal invasion	0.614	0.304	4.094	0.043	1.848	1.019–3.350
Lymph node metastasis	0.577	0.254	5.170	0.023	1.780	1.083–2.927
Tumor location	−0.507	0.205	6.093	0.014	0.602	0.403–0.901

*Analysis of variance. P < 0.05 is significant.

### Impact of independent prognostic factors for survival in patients with cardiac cancer of the remnant stomach and primary cardiac cancer

Subgroup analysis, showed the 5-year survival rates in patients with cardiac cancer of the remnant stomach and primary cardiac cancer were 15.0% and 25.0%, respectively (*P* = 0.041), with serosal invasion; and 10.7% and 21.6%, respectively (*P* = 0.013), with lymph node metastasis. Similarly, the two groups had 5-year survival rates of 25.0% and 43.8%, respectively (*P* = 0.214), without serosal invasion; and 25.0% and 38.8%, respectively (*P* = 0.255), without lymph node metastasis (Table 4).

**Table 4:** Survival rates of patients with cardiac cancer of the remnant stomach and primary cardiac cancer in patients with/without serosal invasion and with/without lymph node metastasis

**Variables**	**Primary cardiac cancer of remnant stomach, *n *** **(%)**	**Primary cardiac cancer, *n *** **(%)**	***X***^**2**^	***P *** **value**
Serosal invasion				
Yes	40 (15.0)	80 (25.0)	4.176	0.041
No	8 (25.0)	16 (43.8)	1.542	0.214
Lymph node metastasis				
Yes	28 (10.7)	56 (21.6)	6.236	0.013
No	20 (25.0)	40 (38.8)	1.294	0.255

*Analysis of variance. P < 0.05 is significant.

### Mortality and complication rates

There was no postoperative death in all patients but the overall postoperative complication rate was 14.6%, 20.8% in patients with cardiac cancer of the remnant stomach, and 11.5% in patients with primary cardiac cancer (*P* = 0.138; Table 5).

**Table 5:** Mortality and complication rates in patients with cardiac cancer of the remnant stomach and primary cardiac cancer

**Variables**	**Primary cardiac cancer of remnant stomach (*n*** **= 48)**	**Primary cardiac cancer (*n*** **= 96)**	***P *** **value**
Mortality	0	0	1.000
Overall complication	10 (20.8)	11 (11.5)	0.138
Surgical complication	5 (10.4)	5 (6.7)	0.255
Anastomotic leak	1 (2.1)	2 (2.1)	
Subdiaphragmatic abscess	0	1 (1.0)	
Anastomotic stricture	2 (4.2)	1 (1.0)	
Abdominal abscess	2 (4.2)	1 (1.0)	
Non-surgical complication	5 (10.4)	6 (6.3)	0.380
Pneumonia	2 (4.2)	3 (3.1)	
Cardiac	0	1 (1.0)	
Abnormal liver function	2 (4.2)	1 (1.0)	
Other infection	1 (2.1)	2 (2.1)	

*Analysis of variance. P < 0.05 is significant.

## Discussion

Cancer of the gastric remnant is defined as a carcinoma detected in the remnant stomach more than 5 years after primary surgery for a benign disease or 10 years after primary surgery for a malignant disease, regardless of the pattern of the initial surgical resection or the type of reconstruction [2,3]. Cardiac cancer of the remnant stomach is defined as a carcinoma in the gastric remnant cancer located in the cardiac region. The treatment of choice for patients with cardiac cancer of the remnant stomach is surgical resection, since aggressive surgical treatment can improve postoperative patient prognosis and quality of life [13]. The prognosis of patients with advanced cardiac cancer of the remnant stomach is poor, with a 5-year survival rate of only 3% to 5% [14]. Earlier diagnosis, a better understanding of the anatomical structure of the remnant stomach, and improvements in surgery have enhanced the 5-year survival rate to as high as 50.9% [15-17]. Although cardiac cancer of the remnant stomach and primary cardiac cancer are both located in the upper third of the stomach, their prognoses have been reported to differ. For example, the survival rates in these patients were reported to be 62.9% and 53.7%, respectively, although the difference was not statistically significant [18]. A study of 15 patients with cardiac cancer of the remnant stomach and 71 with primary cardiac cancer found that the rates of serosal invasion were 40% and 11%, respectively (*P* <0.05), with the 5-year survival rate being significantly lower in the former group [19]. We observed 5-year survival rates of 16.7% and 28.4%, respectively (*P* <0.05). This phenomenon may be related to the biologic nature of the remnant gastric cancer in comparison with primary cancer, there were remarkable differences in the tumor histology of gastric remnant cancer between the early stage mostly having intestinal type and the locally advanced stage having diffuse type [20]. In addition, gastric remnant cancers more frequently showed a poorly differentiated type histology than did upper gastric cancers [21]. Because cardiac cancer of the remnant stomach is often diagnosed at an advanced stage, the likelihood of infiltration of the pancreas, transverse colon, liver, spleen, and other surrounding organs is high. These patients may often require total resection of the remnant stomach, making combined organ resection necessary. In addition, the initial surgery changes the anatomical structure of the remnant stomach, resulting in a change of lymphatic flow [19,22]. Since lymph streams along the posterior gastric artery, splenic artery, and jejunal and colonic mesentery, surgical resection is difficult, and a good effect is hard to achieve [12,23]. Moreover, a high incidence rate of postoperative complications may reduce survival rates [7]. Improvements in surgical technology and perioperative management have greatly improved the safety of the operation, reducing the incidence of postoperative complications [24,25]. We found that the rate of postoperative complications was similar in patients with cardiac cancer of the remnant stomach and those with primary cardiac cancer.

Depth of invasion is an important prognostic factor in patients with cardiac cancer of the remnant stomach. To eliminate its influence, we divided patients into subgroups with and without serosal invasion. Although the 5-year survival rate of patients with cardiac cancer of the remnant stomach and primary cardiac cancer were similar in the absence of serosal invasion, they differed significantly in patients with serosal invasion. In patients without serosal invasion, the tumor is limited to the serosa, with no evidence of cancerous infiltration of surrounding tissues. Surgery may be beneficial in patients with inflammatory tumor adhesion to surrounding tissues and organs. In primary cardiac cancer patients with serosal invasion, a single organ is often involved and the tumor is relatively limited in size; thus combined organ resection can achieve good outcomes. In patients with cardiac cancer of the remnant stomach, the latter may adhere to surrounding organs, allowing cancer cells to infiltrate surrounding tissues and organs by diffusion through the adhesions [26]. Surgical resection rate is low in patients with diffusely infiltrating tumors [27], making patient prognosis worse than in patients with primary cardiac cancer.

Lymph node metastasis is also an important prognostic factor in patients with cardiac cancer of the remnant stomach [24,25]. When lymph node metastases were present, the 5-year survival rate was much lower in patients with cardiac cancer of the remnant stomach than in patients with primary cardiac cancer; in the absence of lymph node metastases, the prognosis in the two groups was equivalent. Previous studies have also shown that the 5-year survival rate in patients with cardiac cancer of the remnant stomach and lymph node metastases, often accompanied by distant metastases, was only 25%, much lower than in patients with primary cardiac cancer and lymph node metastases [6,28]. In the latter patients with regional lymph node metastases, D2 resection could frequently result in complete lymph node dissection. In patients with cardiac cancer of the remnant stomach, however, the initial surgery frequently causes changes in anatomical structure and lymphatic flow in the remnant stomach. Changes in mediastinal lymphatic flow at the gastric fundus and cardia can cause mediastinal lymph node metastasis through the connection between the esophagus and stomach toward the lower esophagus. Cancer cells in patients who have undergone Billroth-II reconstruction can move through the mesentery at the anastomosis of the stomach and jejunum to the lymph nodes at the mesenteric root of the small intestine [29]. Lymph node metastases in patients with cardiac cancer of the remnant stomach are frequently accompanied by distant lymph node metastases at the jejunum, mesenteric root, and mediastinum. This reduces the radical resection rate in these patients, and even extended radical resection was unlikely to have improved patient prognosis.

## Conclusions

Although the survival rates after radical resection in patients with cardiac cancer of the remnant stomach were poorer than in those with primary cardiac cancer, they were similar in survival rates when patients without serosal invasion or lymph node metastasis. Therefore, early detection is an important way to improve overall survival in cardiac cancer of the remnant stomach.

## Competing interests

The authors have no competing interests or financial ties to disclose.

## Authors’ contributions

YW, C-M H, and J-B W conceived of the study, analyzed the data, and drafted the manuscript; C-H Z helped revise the manuscript critically for important intellectual content; PL, J-W X, J-X L, and Jun Lu helped collect data and design the study. All authors read and approved the final manuscript.
